# Environmental Health: Threats and their Interactions

**DOI:** 10.4137/EHI.S1003

**Published:** 2008-11-11

**Authors:** Douglas Holdstock

**Affiliations:** 20 Tanglewood Close, Woking, Surrey GU22 8LG, U.K

**Keywords:** climate change, conflict, environmental health, population growth

## Abstract

Improvements in the provision of an acceptable standard of health care, particularly in the developing world, will be undermined by three ongoing processes: ongoing armed conflicts; the threat of global warming due to rising levels of greenhouse gases, particularly carbon dioxide emitted by developed countries; and by rapidly rising populations. The key features of these three threats are summarised, and it is shown that interactions between them increase both the likelihood of their occurrence and the probable harm that they will cause. Some of the interactions are described, with ways of providing health care taking into account the threats and their interactions, and the paradox is emphasised that better health care in the developing world will further increase population growth followed by increased greenhouse gas emissions. Improved education for women and free and unlimited access to modern methods of contraception are vital.

## Introduction

Throughout human history, the basic human essentials have been adequate food, drinkable water, and adequate shelter. Since the development of modern scientific medicine in the last hundred years or so, a new need is access to health care. Lack of these essentials results in malnutrition leading to starvation and to what are now regarded as preventable or treatable diseases. The provision of these needs is made difficult or even sometimes impossible by three major threats. These are climate change, leading variously to heat stress, drought with crop failure, or flooding; conflict; and overcrowding, either maldistribution of existing populations, as with today’s mega-cities, or population growth outstripping food and water supplies. Moreover, these threats can interact with one another, with positive feedbacks which can precipitate major crises. This paper draws attention to some of these interactions; first, the key features of the threats themselves are summarised.

## Environmental Threats: Climate Change

The climate has always fluctuated, and changes have been linked to disease. The World Health Organization estimates that 150,000 people a year are already dying from the effects of climate change (WHO, 2008).

Today, global warming, now thought by the majority of climate scientists to be due to human emissions of greenhouse gases (Intergovernmental Panel on Climate Change, 2007) threatens health in several ways: drought and crop failures leading to starvation; increasing severity of tropical storms causing flooding, as recently with cyclone Nargis in Myanmar (Burma). Melting of the polar icecaps will raise sea levels, making low-lying areas such as the Pacific islands, and deltas such as that of the Ganges will become uninhabitable (Lynas, 2007).

The basic science of climate change/global warming is clear (Lynas, 2007). The atmosphere transmits sunlight, which warms the earth’s surface; some of this energy is emitted as infra-red radiation. As was first pointed out by the Swedish chemist Arrhenius over a hundred years ago, certain gases, particularly carbon dioxide but also methane, absorb rather than transmit infra-red radiation, heating up the lower atmosphere. Past levels of CO_2_ have kept the surface warm enough to be habitable, but rising levels due to the use of fossil fuels (coal, then oil) in the industrialised societies have resulted in a significant rise in temperature in the past century; observations at Mauna Loa Observatory in Hawaii show a close correlation between the more recent rises in CO_2_ levels and temperature.

The feedbacks could worsen the extent of global warming. Already, melting of the Arctic ice cap is reducing the amount of sunlight reflected back into space. Warming of Siberian permafrost could liberate large amounts of methane, a more potent greenhouse gas than CO_2_ (Lynas, 2007).

## Conflict

The objective of the military and their weapons is to kill (or threaten to kill) an enemy as “efficiently” as possible. War has been endemic in human history, and caused many millions of deaths, some direct, many more indirect among civilians due to disruption of agriculture, trade, and more recently the difficulty of providing health care in war-torn societies. Estimates cannot be accurate, but there are believed to have been over 140 million war-related deaths between 1500 and 1990. The population may have fallen by a third or more in parts of Germany and Eastern Europe during the Thirty Years War (1618–48). Around ten million are thought to have died in World War I and over 50 million in WWII (Holdstock, 2002). Many millions have died in conflict or from its “side-effects” since the end of the Cold War, most in sub-Saharan Africa, especially the Democratic Republic of Congo, and in the Middle East.

Recent research has revealed an unexpected interaction between the human effects of a possible future nuclear war and its climatic consequences. The risk of “nuclear winter” is well-recognised (Turco et al. 1983). New studies on “limited” nuclear exchanges of about 100 Hiroshima-sized weapons (less than 1% of the global stockpile), as could have happened between India and Pakistan over Kashmir not long ago, could generate wildfires in and around major cities. The resulting global cooling could affect agriculture worldwide for up to ten years, causing in turn widespread food shortages, particularly affecting mega-cities. Food riots and epidemics could bring the global death toll up to a billion (Robock et al. 2007; Helfand, 2007).

## Population and Resources

The human population of the world is estimated to have been about three million at the beginning of agriculture in the Fertile Crescent of the Middle East about 10,000 BCE, rising to about ten million in 4000 BCE (Ponting, 2007: p. 37). It rose more rapidly with the start of the Bronze Age and the first cities in Sumeria and the Nile valley, to about 100 million at the BCE/CE transition; the population of Rome at its height may have been about one million.

The Roman empire depended upon trade bringing in corn from outside, particularly from North Africa; both traders and armies returning from colonial wars probably brought back epidemic disease, particularly “Justinian’s plague” in the sixth century CE (perhaps bubonic plague). World population may have fallen from 250 to 200 million from the third to the sixth centuries CE, much of the fall in Europe (Ponting, 2007: pp. 89, 96).

By 1300 CE world population had risen again to about 400 million. However, the Medieval Warm Period had come to an end around 1200 CE (Henson, 2006: p. 210) and was followed by a period of steadily cooling climate, with increasing winter rains leading to water-logging of the soils and reduced crop yields. There was a severe famine in 1316–17. In 1347 the Black Death (bubonic plague) was brought to Europe by Genoese fleeing the Black Sea port of Kaffa (now Feodosia), where it broke out in the Tartars besieging the port. Its spread through Europe has been ascribed o the already poor health of the population, which had reached an optimum for the agricultural technology of the day at the end of the warm period—as had the population of rats which spread the disease (Henson, 2006: p. 147; Ponting, 2008: pp. 96–97). World population fell by about 20 million in the 13th century. Population then rose to about 700 million in 1700, since when it has risen explosively to today’s six and a half billion (Ponting, 2007: pp. 231, 235). This increase is closely related to industrialization, on the back of cheap fossil fuels, first coal and in the twentieth century oil. It has also seen the rise of overcrowded mega-cities where delivery of health care is poor (Ponting, 2008: pp. 302–306). Gang-style conflicts can arise, as publicized by current concern over knife and gun crime in the United Kingdom, but of course also in many parts of the developing world (e.g. Muggah, 2008).

There have been many local but significant fluctuations of population in the past; one familiar to American readers may be that of the Maya in and around the Yucatan in Central America. Relying at first on “swidden” agriculture (progressively slashing and burning the tropical forest for the rotation of crops, including maize), an elaborate civilisation developed, with cities, large pyramids, a written language, astronomy, and a calendar, reaching a peak around 600 CE and dependent upon much more intensive agriculture, with terracing and irrigation. This civilisation collapsed abruptly around 800 CE, perhaps within a few decades, mainly due to unsustainable demand upon the agricultural system, but also with widespread conflict between the cities—as today, armies were regarded as part of the elite of the cities (Webster and Toby Evans, 2005; Ponting, 2007: pp. 78–82).

The problems likely to arise from population growth exceeding resources were first predicted over 200 years ago by Thomas Malthus—when world population was less than one billion (Ponting, 2007: p. 231)—but perhaps most vividly by Ehrlich (1916)—when population had reached about 3.5 billion. Ehrlich’s call for limits to population growth drew only protests at that time. The subject has remained taboo until recently (Hardin, 1993), mainly because the increase in area for agriculture, and increasing agricultural productivity, largely due to fertilisers, has more or less kept pace with the population expansion since Malthus’ times. Today, however, some 800 million people are underfed, and a billion are short of clean water (Rogers, 2008). Compared with the ancient Middle East or Mesoamerica, we have little or no space to move to, or for agriculture to expand, and indeed the effects of climate change, such as drought, are likely to reduce food production (Lynas, 2007).

## Today

Climate change has taken the place of the threat of nuclear war as the current great concern. As noted, the carbon dioxide causing global warming comes largely from the burning of fossil fuels by the developed countries. The United States of America, with 5% of the world’s population, accounts for 25% of global CO_2_ emissions; China has recently overtaken the U.S. as the largest emitter, but with a six times larger population is still far behind in *per capita* emissions. Emissions from India, Brazil and other large middle income and developing countries are also rising rapidly.

The effects of climate change, whether drought or flooding, will include conflict over scarce resources, especially food, and as a result of mass migration. This may already be happening; the basic cause of the conflict in Darfur, with its serious political aspects, may nevertheless be the drought affecting sub-Saharan Africa (Sachs, 2008; pp. 248–253). Drought may also be the underlying cause of the tribal conflicts increasingly affecting the Horn of Africa (Ouyang, 2008; Wakabi, 2008). Sub-Saharan Africa is the region with the most rapid population growth; Rogers (2008) notes the interaction of climate change and population growth in causing a shortage of clean water there.

It is clearly impossible to formally test whether overcrowding (as from rapid population growth) is a cause of conflict in human societies. Forty years ago the Russells described observations of monkeys which do strongly support the hypothesis of overcrowding as a cause of conflict; the zoo societies they discuss were well-fed (Russell and Russell, 1968). The authors, who are regarded as the founders of human ethnology, describe human groups showing analogous behaviour, as do today’s crowded societies, though of course affected by inequalities and injustices. The principal cause of the 1994 genocide in Rwanda was doubtless the long-standing tribal rivalry between the Hutu and the Tutsi. But the genocide occurred in a small landlocked country, which had at that time the highest total fertility rate in the world, about eight children per married couple compared with a replacement level of just over two, with a population already of six million and according to one contemporary observer producing enough to feed only five million (Bligh, 2004). The killing was mainly carried out with machetes; limiting the availability of modern weapons is not enough.

Western military intervention, which today is largely over resources rather than human rights, perpetuates rather than ends conflict. Even its most enthusiastic advocates would surely agree that military action can do nothing positive to prevent climate change or help limit population growth. War indeed is likely to *increase* global warming through the CO_2_ generated by tanks and warplanes, before, during and after wars (Levy and Sidel, 2008). The carbon dioxide released by U.S. forces in the Iraq war equals the emissions from 25 million cars on the U.S. roads in a year; if the war were ranked as a country, it would emit more CO_2_ than 139 of the world’s nations annually, and the U.S. spent more on the war in 2006 than the whole world spent on investment in renewable energy (Reisch and Kretzmann, 2008). By late in 2007 the U.S. government had spent almost $500 billion on the Iraq war and continued to spend more than $2 billion a week (Levy and Sidel, 2008).

Military CO_2_ emissions of course continue even in the absence of war—tank exercises and training flights for warplanes for example. According to the 2005 *Encyclopaedia Britannica* Yearbook (the last time such a figure was quoted) world military expenditure was 2.5% of global Gross Domestic Product. The military effect on climate change could well be higher than this; military jets often fly high in the stratosphere where their vapour trails make a disproportionate contribution to the greenhouse effect (Parkinson, 2007).

## Solutions and the Role of Health Professionals

Environmental health initiatives, such as clean water, sanitation, insecticide-treated bednets, and in the longer term an HIV vaccine, should therefore take climate change, conflict and population growth into account. There is an obvious paradox here; all these initiatives are vital—thirty years after the Alma-Ata Declaration calling for Health for All as a human right, more than nine million children under five years of age are dying every year, more than half of them in sub-Saharan Africa (Loaiza et al. 2008). According to these authors, this is at least in part due to the high fertility rate there, so that improving child survival will increase population pressure. However, when child survival increases, there is clearly less need for more children. A fundamental part of the provisions of adequate healthcare in the developing world, then, is full availability of modern contraceptive advice, with both education for women and, more controversially, access to legalised safe abortion.

Such a programme, especially the latter component, is of course anathema to the Roman Catholic Church, the religious right in the U.S.A. and sections of Islam. But many women will risk the danger of illegal abortion if the legal variety is unavailable. Sachs (2008: pp. 166–170) points out that according to the UN Population Division (2007) a significantly lower total fertility rate could result in world population stabilising at less than eight billion by 2040, followed by a slow fall. In comparison, the UNPD’s widely quoted figure of a global population of nine billion by 2050 is its medium fertility variant; high or constant fertility could lead to still-rising populations of 11 or 12 billion by 2050. It is hard to see how food production could be increased, or distributed even if available, to feed such levels.

Ameliorating or preferably preventing climate change, particularly reducing global carbon dioxide emissions, also raises a paradox: the least developed countries in particular, which currently have very low *per capita* emissions, are bound to increase them. As noted, large developing countries such as China and India are already greatly increasing their CO_2_ emissions, but are refusing to consider limitations unless the developed nations, particularly the U.S.A. promise their own major reductions. Reducing poverty is essential for the provision of adequate health care in areas where it is currently unavailable, but constraints imposed by the threat of global warming imply that poverty cannot be reduced simply by continued economic growth (Woodward and Labonte, 2008).

Of the three threats, the work of health professionals is most directly affected by the rise in population, particularly in Asia and Africa. They are, of course, directly concerned with primary health care, and are also closely involved with providers of clean water and sanitation. They must be aware of the paradox noted above that success in these fields will add further to population growth, and must as part of their work strongly promote free and full access to modern contraceptive care and support wider education for women to allow them to benefit from such care. As also implied above, health workers must also resist any attempts by pressure groups to restrict such access.

Health professionals who are members of the military or closely linked to them must be fully aware of the ethical dilemmas involved, for example in possible interrogation of prisoners. They must remain physicians “first, last, always” (Annas, 2008) and should be prepared to speak out against abuses of human rights. Other doctors and health workers must draw attention to the wider health effects of military intervention, as in Iraq (Roberts et al. 2004), and of the wide availability of small arms in the developing world, often but not always distributed illegally (Muggah, 2008). They should emphasise that such conflicts can continue for many years (Medact, 2008), but also that sanctions too can have serious health effects (Gottstein, 1999). The conclusion is clearly that both sanctions and armed intervention are bad for health, and that conflict must be prevented or resolved through the UN and its agencies.

Doctors and other health professionals can work on climate change individually and locally, and also advocate global action. Locally, they can make their homes energy-efficient and, where available, make use of renewable energy sources. Where possible, they can walk, cycle, or use public transport, and can change to smaller and more energy-efficient cars, and avoid flying as often as possible. They can press for their clinics or hospitals to be energy-efficient, and can call for professional meetings and conferences to be by tele-conferencing whenever possible (Griffiths et al. 2008). More widely, they can draw the attention of the media and decision-makers to the risks from climate change, including the links between climate and other factors—not least that crop failures from drought or floods lead in turn to malnutrition and to mass migration and conflict (Haines et al. 2007; McMichael et al. 2008).

Continued economic growth must surely imply further increases in worldwide emissions of CO_2_ and other greenhouse gases. Various programmes to reduce overall emissions, while still allowing growth in the developing world, have been proposed (Meyer, 2000; Cap and Share, 2008). All sections of the developed countries’ economies must contribute (the “wedge” approach—Pacala and Socolow, 2004; Henson, 2006), even though vested interests such as the oil, aviation and motor industries will plead for exemption. Cuts in military forces and their activities, and in the arms industry that supports them, is surely such a wedge, though one so far little mentioned. The realization that armed violence only leads to more violence, even when committed by “official” forces, should allow such cuts. Keeping armed forces simply as national status symbols is no excuse; their exercises will continue to emit CO_2_. Some wedges are positive: energy conservation and a massive expansion in renewable energy sources will reduce reliance on fossil fuels, and could readily be resourced from cuts in military spending.

Urgent action is essential, and recent studies suggest that we may have as little as ten years to stabilise the climate before an irreversible rise of temperature of four to six degrees centigrade becomes inevitable, as a result of positive feedbacks such as methane liberated from the Siberian tundra (Lynas, 2007). Nor will action be easy. In 2007, it was thought that stabilizing CO_2_ levels at 450 parts per million would be adequate (Hansen et al. 2007; Sachs, 2008; pp. 94–96). However, in their latest paper (Hansen et al. 2008) his group suggests, by comparison with past levels, that the level should be no more than 350 ppm,—significantly *lower* than today’s level of 380 ppm. Governments in both developed and developing worlds will be reluctant to commit to such reductions, particularly at a time of economic crisis when employment is threatened world-wide. They must be reminded (not least by health professionals) that greatly extended work on energy efficiency and on non-nuclear renewable energy sources—including wind, solar, hydrogen, tidal and wave power—will provide plenty of employment for both skilled and less skilled workers. More coal-fired power stations are not an option unless and until carbon capture and storage (CCS) becomes practicable on a large scale. Nor is an expansion in civil nuclear power, not only because of the unsolved problem with disposal of radioactive waste or the risk of diversion of plutonium to military use, but because uranium stocks will become scarce and uneconomic in no more than seventy years’ time (Barnaby, 2008). The 2009 Copenhagen conference to draw up a successor to the Kyoto protocol may give some indication whether the political will for such a programme exists.

## Summary

The provision of health care, particularly in the developing world, is threatened by armed conflicts, climate change due to greenhouse gas emissions, mainly at present from the developed world, and continued world population growth, and by the interactions between these threats. Their features are considered, solutions advocated, and the role of doctors and other health professional in minimising or preferably preventing their effects is discussed.
